# Effects of self-paced interval and continuous training on health markers in women

**DOI:** 10.1007/s00421-017-3715-9

**Published:** 2017-09-20

**Authors:** Luke J. Connolly, Stephen J. Bailey, Peter Krustrup, Jonathan Fulford, Chris Smietanka, Andrew M. Jones

**Affiliations:** 1 Sport and Health Sciences, College of Life and Environmental Sciences, University of Exeter, St Luke’s Campus, Heavitree Road, Exeter, EX1 2LU UK; 2 School of Sport, Exercise and Health Sciences, Loughborough University, Loughborough, UK; 3 Department of Sports Science and Clinical Biomechanics, SDU Sport and Health Sciences Cluster (SHSC), Faculty of Health Sciences, University of Southern Denmark, Odense, Denmark; 4 NIHR Exeter Clinical Research Facility, University of Exeter Medical School, Exeter, UK; 5 Faculty of Sport and Health Sciences, University of St Mark and St John, Plymouth, UK

**Keywords:** Cardiovascular health, Self-paced exercise training, Inactive, Cycling training, Cognitive function, Time commitment

## Abstract

**Purpose:**

To compare the effects of self-paced high-intensity interval and continuous cycle training on health markers in premenopausal women.

**Methods:**

Forty-five inactive females were randomised to a high-intensity interval training (HIIT; *n* = 15), continuous training (CT; *n* = 15) or an inactive control (CON; *n* = 15) group. HIIT performed 5 × 5 min sets comprising repetitions of 30-s low-, 20-s moderate- and 10-s high-intensity cycling with 2 min rest between sets. CT completed 50 min of continuous cycling. Training was completed self-paced, 3 times weekly for 12 weeks.

**Results:**

Peak oxygen uptake (16 ± 8 and 21 ± 12%), resting heart rate (HR) (−5 ± 9 and −4 ± 7 bpm) and visual and verbal learning improved following HIIT and CT compared to CON (*P* < 0.05). Total body mass (−0.7 ± 1.4 kg), submaximal walking HR (−3 ± 4 bpm) and verbal memory were enhanced following HIIT (*P* < 0.05), whereas mental well-being, systolic (−5 ± 6 mmHg) and mean arterial (−3 ± 5 mmHg) blood pressures were improved following CT (*P* < 0.05). Participants reported similar levels of enjoyment following HIIT and CT, and there were no changes in fasting serum lipids, fasting blood [glucose] or [glucose] during an oral glucose tolerance test following either HIIT or CT (*P* > 0.05). No outcome variable changed in the CON group (*P* > 0.05).

**Conclusions:**

Twelve weeks of self-paced HIIT and CT were similarly effective at improving cardiorespiratory fitness, resting HR and cognitive function in inactive premenopausal women, whereas blood pressure, submaximal HR, well-being and body mass adaptations were training-type-specific. Both training methods improved established health markers, but the adaptations to HIIT were evoked for a lower time commitment.

## Introduction

It is well documented that physical inactivity and age are associated with increased morbidity from non-communicable diseases (NCDs) and are accompanied by declining cardiorespiratory fitness, cognitive and metabolic dysfunction, and hypertension (Booth et al. 2012). Middle-aged women are particularly susceptible to develop NCDs and associated co-morbidities, but regular physical activity is known to mitigate the development of NCD risk factors (Garber et al. 2011). Consequently, there are clear physical activity guidelines of 150 min of moderate aerobic activity or 75 min of vigorous aerobic activity per week to improve public health (Garber et al. 2011). Nonetheless, there is evidence that 34–39% of women aged 25–54 years in Western societies such as the United Kingdom are failing to meet physical activity recommendations (Townsend et al. 2015). Therefore, the development of physical activity interventions with high compliance rates are required to improve the health status of inactive middle-aged women.

Regular (2–4 sessions weekly), instructor-directed, continuous (40–60 min), moderate-to-vigorous intensity [55–80% of peak heart rate (%HR_peak_)] exercise has been shown to improve established NCD risk factors in women. Clinically relevant improvements have been reported for peak oxygen uptake ($\dot {V}{{\text{O}}_{{\text{2peak}}}}$, +10–19%) (Kong et al. 2016; Trapp et al. 2008), fat mass (−1.1 to −1.4 kg) (Mohr et al. 2014; Kong et al. 2016), systolic blood pressure (BP) (−4 to −6 mmHg) (Mohr et al. 2014), resting heart rate (HR) (−5 bpm) (Mohr et al. 2014), fasting blood glucose (−5%), [insulin] (−28%) and insulin sensitivity (+27%) (Ciolac et al. 2010; Robinson et al. 2015) and plasma [low-density lipoprotein] (LDL) (−5%) and [high-density lipoprotein] (HDL) (+3%) (Kelley et al. 2004). While regular regimented CT training can attenuate the development of NCDs, this fixed intensity training regimen requires a significant weekly time commitment. Since time constraints and enjoyment are commonly cited as two of the main barriers preventing middle-aged women from meeting physical activity guidelines (Booth et al. 2012), the time commitment of CT training might contribute to the large percentage of middle-aged women who fail to meet physical activity guidelines (Townsend et al. 2015).

In contrast, high-intensity interval training (HIIT) may provide an alternative, more time-efficient strategy for improving the health profile of inactive premenopausal women. Indeed, instructor-directed HIIT has been shown to improve cardiorespiratory fitness (Trapp et al. 2008; Trilk et al. 2011; Weston et al. 2014), body composition (Mohr et al. 2014; Trapp et al. 2008), resting systolic BP and HR (Mohr et al. 2014), and fasting blood [glucose], [insulin] and insulin sensitivity (Connolly et al. 2016; Trapp et al. 2008) to the same or greater extent compared to CT. However, some of these training-induced responses to HIIT, in particular, those related to insulin sensitivity, have been reported to be blunted in women compared to men (Gibala et al. 2014) and some HIIT regimens comprise repeated ‘all-out’ sprints, which may be unsuitable for inactive middle-aged women. It is therefore unsurprising that HIIT data on middle-aged women and women in general is limited (Weston et al. 2014). In addition, the majority of studies investigating the effects of HIIT on health outcomes have imposed longer intervals or strict work rates allowing minimal variation in session intensity which may adversely impact participant enjoyment and HIIT adherence (Kong et al. 2016; Trapp et al. 2008). Unfortunately, studies related to HIIT for women have not reported data regarding enjoyment (Mohr et al. 2014; Connolly et al. 2016; Trapp et al. 2008; Trilk et al. 2011). As a result, it is unclear whether HIIT is more or less enjoyable compared to CT (Bartlett et al. 2011; Foster et al. 2015).

It has been reported that interventions aimed at increasing physical activity tend to be more effective when the intensity of physical activity is lower rather than higher (Dishman and Buckworth 1996). However, evidence suggests that novice exercisers are generally inaccurate in self-monitoring and self-regulating the intensity of their efforts when the intensity is prescribed (Duncan et al. 2001). For example, it has been reported that women exceed their target heart rates when participating in aerobics, resulting in exercise intensities much higher than expected (Laukkanen et al. 2001). However, despite this, very little is presently known about how the exercise intensity chosen by previously inactive middle-aged premenopausal women may change over the course of a prolonged HIIT and CT intervention.

Cognitive function has been shown to decline in middle-aged adults (45–49 years) (Singh-Manoux et al. 2012) and it is known that participation in exercise can alleviate this decline in older individuals (Angevaren et al. 2007). There is some evidence to suggest that the acute improvement in cognitive function after an exercise bout is more pronounced following short-duration HIIT exercise compared to CT with this effect attributed to enhanced cerebral blood flow in the former compared to the latter (Angevaren et al. 2007). However, at present, it is not clear if chronic HIIT and CT would have different effects on cognitive function in inactive middle-aged women. Consequently, further research is required to assess how self-paced HIIT and CT influence enjoyment, training progression (increases in work rate during training sessions), adherence, and established health markers in inactive middle-aged premenopausal women.

The aim of the present study was therefore to investigate the effects of 12 weeks of self-paced HIIT and CT training on cardiorespiratory fitness, BP, parameters of cognitive function, blood glucose tolerance, serum lipid profile, body composition, and enjoyment of and adherence to exercise in inactive premenopausal women. It was hypothesised that both self-paced training methods would evoke beneficial changes in the health profile parameters outlined above, but that self-paced HIIT would elicit equivalent physiological and greater cognitive adaptations for a lower time commitment compared to self-paced CT training.

## Methods

### Subjects

Participants were recruited through advertisements in local community venues and the University of Exeter news bulletin. All participants gave their written informed consent after being informed verbally and in writing of the experimental procedures, potential benefits, and the possible risks and discomforts associated with the study. Individuals expressing an interest in participating completed a questionnaire to confirm that they met the inclusion criteria of being premenopausal, non-smokers, not pregnant or on medication, and without known metabolic or cardiovascular diseases. It was also confirmed that none of the participants had ever been an athlete or participated in vigorous exercise and were currently inactive, having not participated in regular physical activity for at least 2 years. The participants completed the International Physical Activity Questionnaire. Participants self-reported their dietary intake during the first, sixth and last week of the intervention period which was subsequently analysed by a member of the research team using nutritional software (Nutritics v4.267 Academic Edition, Dublin, Ireland). Food records were analysed to assess the type and amount of food consumption. Participants were also encouraged to avoid deviation from their normal dietary practices for the duration of the study and to maintain their normal lifestyle. The study was approved by the Sport and Health Sciences Research Ethics Committee at the University of Exeter, Exeter, UK.

### Experimental design

The study was designed as a randomised controlled trial. Fifty-five women were initially recruited; however, seven failed to meet the inclusion criteria and were, therefore, excluded from the study. Once inclusion criteria were met, participants were randomly assigned to a high-intensity interval training cycling group (HIIT: age, 44 ± 7 (mean ± SD) years; height, 1.63 ± 0.04 m; body mass, 67.3 ± 13.5 kg; *n* = 16), a continuous cycling group (CT: age, 43 ± 7 years; height, 1.64 ± 0.07 m; body mass, 72.6 ± 17.5 kg; *n* = 16), or a control group (CON: age, 45 ± 7 years; height 1.63 ± 0.09 m; body mass, 72.0 ± 17 kg; *n* = 16). One participant from HIIT and CT withdrew from the study due to non-study related injuries (lower back and knee pain) and one participant from CON withdrew due a substantial increase in physical activity. Of the 45 participants who completed the study, those in HIIT and CT completed a 12-week training programme as described below, while CON continued their normal daily lives. Before and after the 12-week intervention period, participants completed a series of tests in the laboratory consisting of two separate visits both pre and post intervention.

### Training

The exercise training interventions consisted of an indoor cycling program with exercise sessions completed 3 times per week for 12 weeks. All sessions were preceded by a 5 min warm up at 50 W and a 5 min cool down at 50 W, and were supervised by members of the Sport and Health Sciences department at the University of Exeter for safety reasons. The training sessions were organised around the availability of each individual participant and ranged from 1 to 5 participants cycling at any one time. As the cycle ergometers were moveable, participants had the choice to cycle as a group or independently. Audio and visual entertainment were provided. The training sessions were completed on a cycle ergometer (Monark 894E, Monark Exercise AB, Sweden) which was interfaced with Wingate Anaerobic Test software (Monark Exercise AB, Sweden) to record power output (W) and work done (kJ). HR (Polar RS400, Polar Electro Oy, Kempele, Finland) and ratings of perceived exertion (RPE, 10-point scale) were recorded during every training session. HR data were subsequently downloaded using Polar ProTrainer 5 (Polar Electro Oy, Kempele, Finland) and mean session HR was calculated. %HR_peak_ was calculated from values recorded during an incremental cycling test to exhaustion.

**Fig. 1:**
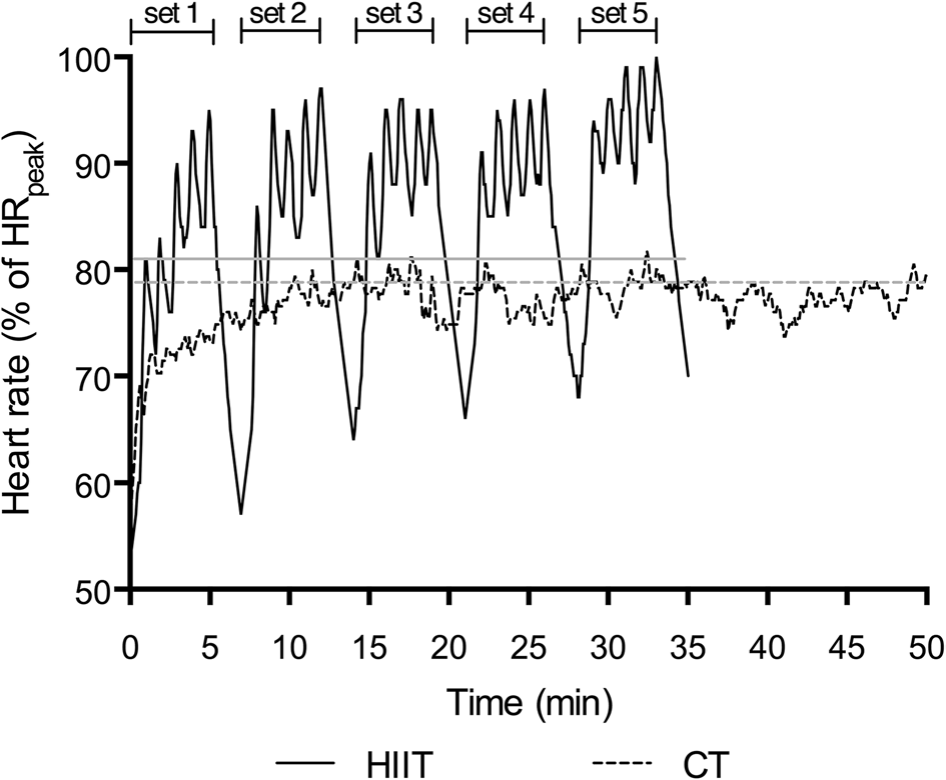
Actual heart rate during a single training session for a single participant from the high-intensity interval training (HIIT) group and the continuous training (CT) group represented by black lines. Grey lines represent mean session HR for HIIT and CT

The HIIT cycling protocol was a modification of the “10-20-30” running training program of Gunnarsson and Bangsbo (2012). Briefly, each participant completed repeated 1 min self-paced exercise bouts comprising 30 s low-intensity (~30% of maximum effort), 20 s moderate-intensity (~50–60% of maximum effort) and 10 s high-intensity (>90% maximum effort) cycling. This 1-min cycle was repeated for 5 min with each 5 min block separated by 2 min passive recovery. During the first week of training (familiarisation), participants were asked to perform 3–4 × 5 min bouts. In subsequent weeks participants completed 5 × 5 min bouts interspersed with 2 min recovery (Fig. 1). Effective training time equated to 25 min per session.

The CT protocol consisted of cycling continuously at a self-paced intensity which they could sustain for 50 min. During the first week of training (familiarisation), participants were asked to cycle continuously for 30–40 min. In subsequent weeks, participants cycled continuously for 50 min each session (Fig. 1). Effective training time equated to 50 min per session.

During the first training session, participants were given guidance on the requirements of each protocol but the intensities during all training sessions were self-selected by the individual and self-adjusted throughout the intervention period. A higher work rate could be attained by an individual self-selecting to increase pedal cadence and/or the resistance applied to the flywheel. The HIIT group were supplied with a timer to remind participants when to switch exercise intensity. Participants were able to visualise in real-time their revolutions per min trace on the cycle ergometer’s electronic display which may have helped maintain the specific exercise intensities.

### Cognitive testing

During cognitive testing participants completed verbal and attention/working learning and memory tasks using the Cogstate computerised cognitive testing system (Cogstate Ltd., Melbourne, VIC, Australia). These parameters were chosen as they have been connected with age-related cognitive decline, mobility and the ability to perform daily activities but have also been reported to improve following exercise (Cox et al. 2015). The specific tests employed consisted of the One Card Learning Task (OCL, 6 min), One Back (ONB, 4 min) and Two Back Task (TWOB, 4 min), and the International Shopping List Task (ISLT, 5 min) including delayed recall (ISLTR, 2 min). The primary performance measure for OCL, ONB and TWOB task was the proportion of correct answers (accuracy) which was normalised using an arcsine square-root transformation. A higher score equated to a better performance. The primary outcome measure for ISLT was the total number of correct responses when remembering a shopping list over three repetitions while ISLTR assessed the shopping list recall following a delay. A higher score equated to a better performance. The order of cognitive function tests was standardised and the total time to complete all tests was around 21 min.

### Experimental testing

Participants were required to refrain from alcohol and exercise for 48 h preceding all visits and instructed to report to the laboratory at least 2 h postprandial in a rested state. On arrival at the laboratory for the first visit pre intervention, participants were familiarised to the cognitive function tests that would occur during the second visit pre and post intervention (see below). Height (Seca stadiometer SEC-225; Seca, Hamburg, Germany), body mass (Seca digital column scale SEC-170, Seca, Hamburg, Germany), body mass index (BMI) and waist-to-hip ratio were obtained prior to testing. Thereafter, pulmonary gas exchange (Cortex Metalyzer 3B, Leipzig, Germany) and HR were measured during the final 3 min of a standardized treadmill test (Woodway Desmo, Waukesha, WI, USA), consisting of 6 min walking at 4 km h^−1^ with 1% incline, and throughout a standardised incremental cycle test to exhaustion (Lode Excalibur Sport V2 electrically-braked cycle ergometer, Lode BV, Groningen, The Netherlands). The ramp incremental test commenced with 3 min cycling at 20 W followed by a continuous linear increase in work rate of 15 W min^−1^. The ramp test ceased when cadence dropped >10 rev min^−1^ below the prescribed cadence of 70 rpm. Along with vocal encouragement to continue to exhaustion, the criteria used to determine $\dot {V}{{\text{O}}_{{\text{2peak}}}}$ included a plateau in $\dot{\mathrm{V}}$O_2_ toward the end of the test despite an increase in workload, a respiratory exchange ratio value ≥1.10 and a HR_peak_ within five beats of a participant’s age-related estimated HR_peak_. Participants gave a symptom-limited maximal effort on all occasions. $\dot {V}{{\text{O}}_{{\text{2peak}}}}$ and HR_peak_ were determined as the peak value reached in a 30-s period during the incremental test.

The second visit pre and post intervention was undertaken no less than 48 h following the first visit pre or post intervention and ultimately no less than 96 h following the final exercise training session. Following an overnight fast and under standardised conditions at ∼08:00, resting HR and BP were measured following the participant sitting quietly for 10 min. BP was measured five times using a semi-automated device (Dinamap Pro 100V2, GE Medical Systems Information Technologies 2002, Tampa, Florida, USA) and the mean of the final three measurements was used to determine resting systolic and diastolic BP. Rate pressure product (RPP) was calculated as HR × systolic BP and mean arterial pressure (MAP) was calculated as 1/3 × systolic pressure + 2/3 × diastolic pressure. Subsequently a 4 ml blood sample was drawn from an antecubital vein into serum separator tubes (Vacutainer, Becton–Dickinson, NJ, USA) and left to clot for 45 min at room temperature. Samples were then centrifuged at room temperature at 1300 relative centrifugal force for 10 min and serum supernatants were removed and stored at −80 °C for later analysis. Samples were analysed using an automatic analyser (Roche Modular P-module, Roche Diagnostics, Indianapolis, IN) for [HDL] (coefficient of variation (CV) 2.1%), total [cholesterol] (CV 2.3%) and [triglycerides] (CV 2.4%). [LDL] was derived using the Friedewald formula (Friedewald et al. 1972).

Following HR, BP and blood measurements, the cognitive battery was undertaken. Thereafter, body composition (body fat percentage, total fat mass and total lean mass) was measured using air displacement plethysmography (The BodPod Body Composition System, Life Measurement Instruments, Concord, CA, USA). Participants then provided a capillary blood sample for assessment of fasting blood [glucose] (YSI 2300 glucose/lactate analyser, Yellow Springs Instruments, Kent, UK) and [haemoglobin] (HemoCue HB 201, Angelholm, Sweden). After consuming 75 g glucose in 300 ml of water, capillary blood samples were collected at 30, 60, 90 and 120 min for the assessment of blood [glucose] and total area under the curve (tAUC) (GraphPad Prism, San Diego, CA). Following the OGTT, subjective mental well-being was measured using the 14-item Warwick-Edinburgh Mental Well-being Scale (WEMWBS) (Tennant et al. 2007). For the post intervention visit, enjoyment of the exercise intervention was also assessed using a modified version of the Groningen Enjoyment Questionnaire (GEQ) (Stevens et al. 2000).

### Statistical analysis

Statistical analyses were performed using the Statistical Package for the Social Sciences (SPSS v21, SPSS Inc., Chicago, IL, USA). A two-factor mixed analysis of variance (ANOVA) design with the between factor ‘group’ (HIIT versus CT versus CON) and the repeated factor ‘time’ (pre-intervention versus post-intervention) was used to analyse all data. Data recorded during training sessions were clustered into 4-week periods and a two-factor mixed ANOVA with the between factor ‘group’ (HIIT versus CT) and repeated factor ‘time’ (1–4, 5–8 and 9–12 weeks) used to analyse training session variables (i.e. HR, RPE, power output and total work done). Effect size was calculated using partial Eta squared ($\eta _{{{\text{partial}}}}^{2}$) which ranged from very small (<0.01) to small (0.01–0.05) to medium (0.06–0.13) to large (≥0.14) (Cohen 1988). Where the effect of the intervention was shown to be statistically significant, all post hoc comparisons were Bonferroni adjusted. The significance level was *P* < 0.05. Data are reported as mean ± standard deviation (SD).

## Results

### Training data

Diet was stable for HIIT, CT and CON when analysed at baseline, 6 weeks and 12 weeks of the intervention (Table 1). Both HIIT and CT groups completed a total of 35 ± 1 training sessions over the 12-week period, corresponding to 2.9 ± 0.1 sessions per week. Weekly training time spent performing HIIT (75 min/week) was half that of the time spent performing CT (150 min/week). Mean power output during the training sessions increased from 1–4 to 5–8 to 9–12 weeks in both groups (*P* < 0.01) with no difference between groups (*P* > 0.05), but CT completed more work than HIIT at all time points (*P* < 0.01, Table 2). Mean HR was not different between training groups during the intervention (*P* > 0.05, Table 2). For the HIIT group, the %HR_peak_ achieved in the 10 s high-intensity periods during weeks 1–4, 5–8 and 9–12 was 94 ± 2, 94 ± 1 and 95 ± 3%. RPE was higher in weeks 1–4 and 5–8 (*P* < 0.05), but not weeks 9–12 (*P* > 0.05) in HIIT compared to CT (Table 2).

**Table 1:** Dietary intake during weeks 1, 6 and 12

	HIIT (*n* = 15)			CT (*n* = 15)			CON (*n* = 15)			Interaction effect	
	Week 1	Week 6	Week 12	Week 1	Week 6	Week 12	Week 1	Week 6	Week 12	*P*	Partial *η* ^2^
Energy intake (kcal)	2009 ± 315	2020 ± 314	2033 ± 307	2043 ± 308	2058 ± 304	2042 ± 296	2136 ± 362	2147 ± 279	2018 ± 306	0.087	0.091
Energy from CHO (%)	53 ± 9	51 ± 9	54 ± 7	50 ± 8	52 ± 7	52 ± 6	53 ± 7	56 ± 8	53 ± 5	0.489	0.039
Energy from fat (%)	30 ± 5	31 ± 6	31 ± 5	33 ± 5	33 ± 5	32 ± 4	32 ± 4	30 ± 5	32 ± 4	0.596	0.032
Energy from protein (%)	17 ± 6	17 ± 6	16 ± 4	18 ± 5	15 ± 5	16 ± 4	16 ± 5	14 ± 6	15 ± 3	0.703	0.025

Values are expressed as means ± SD

Partial *η* ^2^ value for effect sizes

*P* values for interaction (group × time) effect

*CHO* carbohydrate, *CT* continuous training group, *CON* control group,* HIIT* high-intensity interval training group

**Table 2:** Mean training data during intervention

	HIIT (*n* = 15)			CT (*n* = 15)			Interaction effect	
	1–4 weeks	5–8 weeks	9–12 weeks	1–4 weeks	5–8 weeks	9–12 weeks	*P*	Partial *η* ^2^
Power output (W)	95 ± 20	105 ± 17^†^	109 ± 18^†$^	81 ± 16	94 ± 18^†^	100 ± 20^†$^	0.203	0.057
Heart rate (%HR_peak_)	81 ± 4	81 ± 5	81 ± 3	78 ± 5	79 ± 5	79 ± 6	0.587	0.019
RPE (0–10 scale)	6.1 ± 1.2	5.8 ± 1.3	5.2 ± 1.2	4.5 ± 1.5*	4.6 ± 1.7*	4.7 ± 1.7	0.003	0.212
Total work done (kJ)	146 ± 25	149 ± 49	153 ± 28	247 ± 46*	278 ± 55^†^*	286 ± 55^†^*	0.031	0.117
Weekly training time commitment	~75 min (~129 min including warm up, rest and cool down)			~150 min (~180 min including warm up and cool down)				

Values are expressed as means ± SD

Partial *η* ^2^ value for effect sizes

*P* values for interaction (group × time) effect

*CT* continuous training group, *HIIT* high-intensity interval training group, *HR*_*peak*_ heart rate peak (includes recovery periods for HIIT group), *RPE* rating of perceived exertion

*Significantly different from HIIT at same time point (*P* < 0.01)

^†^Significantly different from 1 to 4 weeks

^$^Significantly different from 5 to 8 weeks

### Body composition

Body composition was not significantly different between groups at baseline. Body mass index, body fat percentage, lean mass, fat mass, and waist-to-hip ratio were not different when comparing post- to pre- intervention following HIIT, CT or CON (*P* > 0.05); however, total body mass was lower following HIIT (−0.7 ± 1.4 kg, *P* < 0.05, Table 3).

**Table 3:** Outcome measures pre and post 12-week intervention

	HIIT (*n* = 15)		CT (*n* = 15)		CON (*n* = 15)		Interaction effect	
	Pre	Post	Pre	Post	Pre	Post	*P*	Partial *η* ^2^
TBM (kg)	67.3 ± 13.5	66.6 ± 13.7^†^	72.6 ± 17.5	72.1 ± 17.8	76.1 ± 19.4	76.5 ± 19.0	0.066	0.122
BMI (kg/m^2^)	25.3 ± 4.9	25.0 ± 5.1	26.9 ± 6.3	26.8 ± 6.4	28.4 ± 6.9	28.5 ± 6.6	0.130	0.093
LM (kg)	42.5 ± 4.2	42.3 ± 4.1	42.9 ± 6.2	42.6 ± 6.2	44.1 ± 6.8	44.1 ± 6.6	0.899	0.005
FM (kg)	24.3 ± 11.6	24.0 ± 13.2	29.2 ± 14.6	29.3 ± 15.2	31.3 ± 15.9	31.0 ± 15.7	0.892	0.005
BF (%)	35.0 ± 9.1	33.9 ± 10.0	37.9 ± 9.7	37.7 ± 9.6	37.3 ± 10.4	37.1 ± 10.1	0.431	0.039
Waist-to-hip ratio	0.84 ± 0.06	0.82 ± 0.04	0.81 ± 0.06	0.82 ± 0.04	0.85 ± 0.06	0.86 ± 0.05	0.290	0.057
$$\dot {V}{{\text{O}}_{{\text{2peak}}}}$$								
l·min^−1^	1.71 ± 0.26	1.92 ± 0.27^†^	1.76 ± 0.35	2.09 ± 0.32^†^*	1.71 ± 0.27	1.77 ± 0.29	<0.001	0.411
ml·kg^−1^·min^−1^	26.1 ± 5.9	30.2 ± 6.5^†^	24.8 ± 4.8	29.7 ± 5.0^†^	25.3 ± 4.6	25.7 ± 4.5	<0.001	0.523
Resting systolic BP (mmHg)	111 ± 15	109 ± 14	112 ± 6	107 ± 7^†^	116 ± 17	116 ± 16	0.026	0.160
Resting diastolic BP (mmHg)	69 ± 9	69 ± 9	70 ± 5	68 ± 8	71 ± 9	72 ± 7	0.593	0.025
MAP (mmHg)	83 ± 10	83 ± 10	84 ± 5	81 ± 7^†^	86 ± 12	86 ± 11	0.249	0.064
Resting HR (bpm)	70 ± 6	65 ± 8^†^	67 ± 6	63 ± 7^†^	68 ± 8	69 ± 7	0.115	0.098
RPP	7754 ± 1110	7184 ± 1393^†^	7555 ± 730	6760 ± 775^†^*	7982 ± 1745	7989 ± 1691	0.025	0.161
Fasting blood glucose (mmol/L)	4.17 ± 0.53	4.26 ± 0.48	4.14 ± 0.37	4.07 ± 0.53	3.58 ± 0.84	3.84 ± 0.56	0.283	0.058
tAUC (mmol/L*120 min)	704 ± 88	732 ± 126	695 ± 108	700 ± 105	662 ± 187	666 ± 175	0.682	0.018
Hb (g/L)	134 ± 7	131 ± 13	133 ± 11	133 ± 12	135 ± 7	134 ± 6	0.474	0.035
TC (mmol/L)	4.50 ± 0.70	4.65 ± 0.83	4.85 ± 0.67	4.71 ± 0.57	5.13 ± 1.06	5.18 ± 1.02	0.292	0.580
HDL (mmol/L)	1.77 ± 0.36	1.73 ± 0.39	1.87 ± 0.42	1.80 ± 0.40	1.34 ± 0.53	1.34 ± 0.50	0.540	0.029
LDL (mmol/L)	2.40 ± 0.77	2.52 ± 0.84	2.68 ± 0.50	2.60 ± 0.44	3.13 ± 1.19	3.19 ± 1.15	0.303	0.055
TCl-HDL ratio	2.65 ± 0.60	2.73 ± 0.59	2.69 ± 0.54	2.75 ± 0.58	3.11 ± 1.44	3.20 ± 1.41	0.917	0.004
Triglycerides (mmol/L)	0.82 ± 0.31	0.87 ± 0.44	0.68 ± 0.26	0.73 ± 0.28	0.91 ± 0.32	0.90 ± 0.30	0.886	0.006
Submaximal $\dot{\mathrm{V}}$O_2_								
ml·kg^−1^·min^−1^	11.4 ± 1.5	10.6 ± 1.2	10.9 ± 2.3	10.7 ± 2.3	11.2 ± 2.1	11.2 ± 1.9	0.089	0.111
l·min^−1^	0.75 ± 0.18	0.71 ± 0.19	0.77 ± 0.24	0.76 ± 0.22	0.81 ± 0.19	0.82 ± 0.18	0.180	0.080
Submaximal HR (%HR_max_)	55 ± 7	52 ± 6^†^	55 ± 6	54 ± 8	55 ± 10	57 ± 11	0.063	0.123
WEMWBS	52 ± 9	54 ± 7	49 ± 9	52 ± 7^†^	42 ± 8	42 ± 6	0.198	0.074
GEQ		56 ± 8		53 ± 10				

Vales are expressed a means ± SD. *P* values for interaction (group × time) effect

Partial η^2^ value for effect sizes

*BF* body fat, *BMI* body mass index, *BP* blood pressure, *CON* control group, *CT* continuous training group, *FM* fat mass, *GEQ* Groningen Enjoyment Questionnaire, *Hb* haemoglobin, *HDL* high density lipoprotein cholesterol, *HIIT* high-intensity interval training group, *HR* heart rate, *LDL* low density lipoprotein cholesterol, *LM* lean mass, *MAP* mean arterial pressure, *RPP* rate pressure product, *tAUC* total area under the curve, *TBM* total body mass, *TC* total cholesterol, $\dot {V}{{\text{O}}_{{\text{2peak}}}}$ peak oxygen uptake, *WEMWBS* Warwick-Edinburgh Mental well-being scale

*Significantly different from CON (*P* < 0.05)

^†^Significantly different versus pre-training (*P* < 0.05)

### Exercise variables

**Fig. 2:**
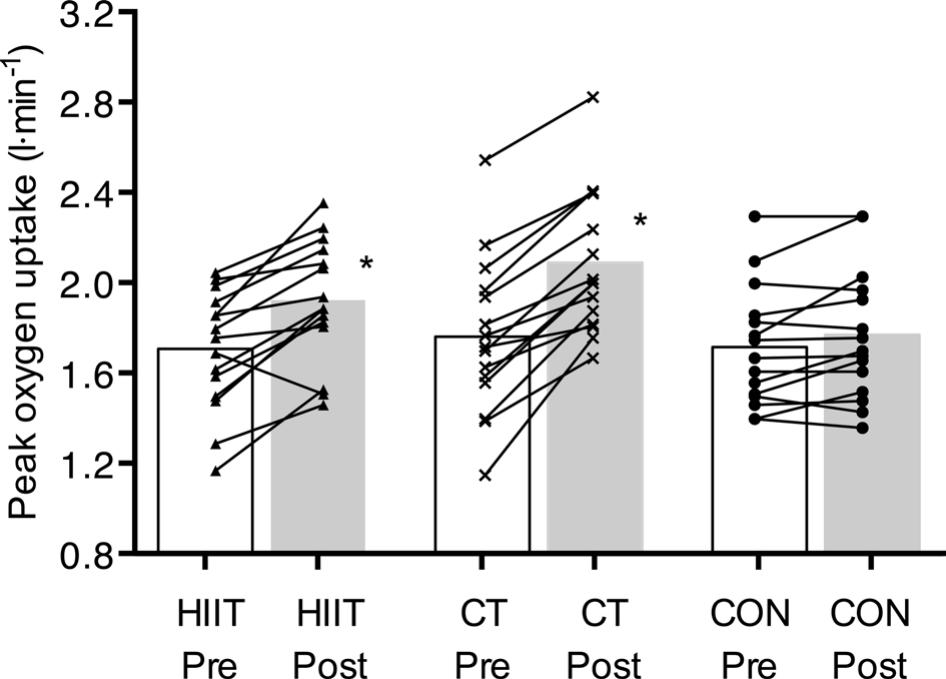
Peak oxygen uptake (l·min^−1^) before and after 12 weeks of high intensity interval training (HIIT), continuous training (CT) and continuation of an inactive lifestyle (CON). Data are mean ± SD. Asterisks denotes significant difference from pre intervention. *P* < 0.05

HR during the submaximal treadmill walking test was lower when comparing post intervention to pre- after HIIT (*P* < 0.05) but not CT or CON (*P* > 0.05, Table 3). Submaximal $\dot{\mathrm{V}}$O_2_ (absolute and relative to body mass) was not different following HIIT, CT or CON (*P* > 0.05). There was a significant group × time interaction for $\dot {V}{{\text{O}}_{{\text{2peak}}}}$ (absolute and relative to body mass, *P* < 0.01) during the ramp incremental cycling test to exhaustion, with post hoc tests revealing a 16 ± 8% (*P* < 0.01) and 21 ± 12% (*P* < 0.01) improvement following HIIT and CT (Fig. 2) but not CON, respectively. There was also a group × time interaction for peak power output (PPO) during the ramp incremental test to exhaustion (*P* < 0.05). PPO was higher post training compared to pre training for HIIT and CT (*P* < 0.05) with no pre-post difference in the CON group (*P* > 0.05). There were no differences between training groups for any of the exercise variables (*P* > 0.05).

### Resting BP and HR

Resting BP and HR were not significantly different between groups at baseline. There was a group × time interaction for systolic BP (*P* < 0.05). Post hoc tests revealed a reduction in systolic BP was apparent following CT (*P* < 0.05) but not HIIT or CON (*P* > 0.05, Table 3). Diastolic BP was not different following CT, HIIT or CON (*P* > 0.05); however, MAP was lower after CT (P = 0.02), but not HIIT or CON (*P* > 0.05). Resting HR and RPP were lowered following HIIT and CT but not CON (*P* > 0.05).

### Serum lipids, haemoglobin and blood glucose

**Fig. 3:**
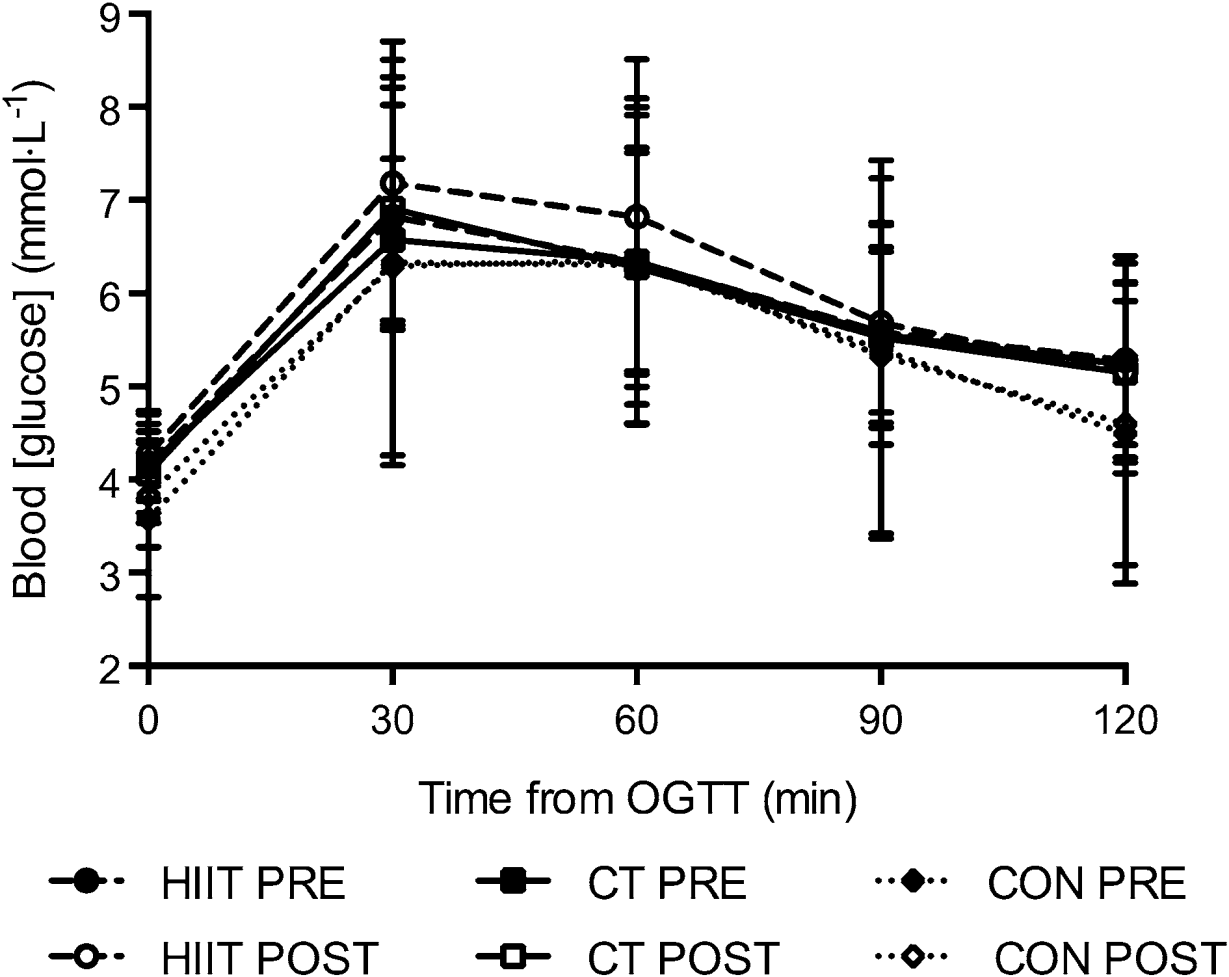
Blood [glucose] response during the oral glucose tolerance test (OGTT) displayed over time for the high-intensity interval training (HIIT) group, continuous training (CT) group and the control (CON) group before and after the 12-week intervention. Data are mean ± SD

Serum total cholesterol-HDL ratio, total [cholesterol], [HDL], [LDL], [triglycerides], [haemoglobin], and fasting and OGTT [glucose] were unchanged when comparing post intervention to pre- for HIIT, CT or CON (*P* > 0.05, Table 3; Fig. 3).

### Mental well-being and enjoyment

WEMWBS scores increased following CT (*P* < 0.05, Table 3) but not HIIT or CON. There was no difference in WEMWBS between training groups. GEQ was not different between the two training groups following the intervention (*P* > 0.05, Table 3).

### Cognitive function tests

Performance was enhanced in the OCL, ISLT and ISLTR tests following HIIT (*P* < 0.05, Table 4) representing an improvement in visual learning and memory, and verbal learning and memory, respectively. Performance was improved in the OCL and ISLT tests after CT (*P* < 0.05, Table 4) representing an improvement in visual learning and memory and verbal learning, respectively. No changes were observed for CON. There were no significant differences between training groups for any cognitive function tests.

**Table 4:** Cognitive function test scores pre and post 12-week intervention

	HIIT (*n* = 15)		CT (*n* = 15)		CON (*n* = 15)		Interaction effect	
	Pre	Post	Pre	Post	Pre	Post	*P*	Partial *η* ^2^
OCL (acc)	0.98 ± 0.18	1.09 ± 0.10*^†^	1.03 ± 0.10	1.09 ± 0.08*^†^	1.02 ± 0.13	1.00 ± 0.13	0.007	0.211
ONB (acc)	1.35 ± 0.19	1.39 ± 0.14	1.32 ± 0.17	1.44 ± 0.12	1.34 ± 0.22	1.33 ± 0.21	0.138	0.090
TWOB (acc)	1.26 ± 0.21	1.33 ± 0.14	1.30 ± 0.17	1.33 ± 0.15	1.19 ± 0.27	1.20 ± 0.29	0.407	0.042
ISLT (cor)	28.5 ± 3.4	30.3 ± 3.2*^†^	30.3 ± 2.8	32.1 ± 1.9*^†^	28.8 ± 3.4	27.9 ± 2.9	0.001	0.283
ISLTR (cor)	9.9 ± 2.3	10.7 ± 1.3^†^	10.9 ± 1.0	11.3 ± 1.0*	10.0 ± 1.9	10.0 ± 1.8	0.053	0.130

Vales are expressed a means ± SD

*P* values for interaction (group × time) effect. Partial *η* ^2^ value for effect sizes

*Acc* proportion of correct answers (accuracy) normalised using an arcsine square-root transformation, *CON* control group, *cor* total number of correct responses to remembering a shopping list, *CT* continuous training group, *HIIT* high-intensity interval training group, *ISLT* international shopping list task, *ISLTR* International shopping list recall, *OCL* one card learning, *ONB* one back memory, TWOB: two back memory

*Significantly different from CON (*P* < 0.05)

^†^Significantly different versus pre-training (*P* < 0.05) as determined by post-hoc analyses following a group × time interaction

## Discussion

The main original findings of the present study were that self-paced HIIT and CT were both effective at improving $\dot {V}{{\text{O}}_{{\text{2peak}}}}$, resting HR, RPP and cognitive function of previously inactive middle-aged premenopausal women. A novel feature of our study was the self-paced nature of the training which might be considered to have better ecological validity than instructor-directed exercise in laboratory studies. Interestingly, as reflected by training HR, the CT group sustained a vigorous intensity for 50 min which would exceed physical activity recommendations (Garber et al. 2011). The similar mean HR during training may also help explain the similar adaptations between HIIT and CT.

The training interventions also resulted in comparable levels of adherence and enjoyment, but the adaptations to HIIT were achieved despite completing 49, 62 and 61% less work (kJ) in weeks 1–4, 5–8 and 9–12 and committing less time compared to CT training. However, while HIIT and CT training resulted in some common improvements in NCD risk factors, a reduction in BP and an increase in well-being were only apparent following CT training whereas improvements in verbal, memory and reductions in submaximal exercise HR and total body mass were only found following HIIT. Therefore, while self-paced HIIT and CT can equally improve several parameters related to the health profile of previously inactive middle-aged premenopausal women, HIIT was a more time efficient strategy to induce such changes. These findings suggest that self-paced exercise has the potential to improve the health profile of inactive middle-aged premenopausal women which might have important implications for future exercise prescription.

The present study demonstrated that 3 weekly HIIT and CT cycling training sessions over 12 weeks resulted in a 16% and 21% improvement in $\dot {V}{{\text{O}}_{{\text{2peak}}}}$, respectively, with no difference between the training groups. Importantly, the reported improvements in $\dot {V}{{\text{O}}_{{\text{2peak}}}}$ (+4.1 ml·kg^−1^·min^−1^ following HIIT and +4.9 ml·kg^−1^·min^−1^ following CT training) are clinically relevant as a 3.5 ml·kg^−1^·min^−1^ increase in exercise capacity relates to a 17% reduction in all-cause mortality (Gulati et al. 2003). The increase in $\dot {V}{{\text{O}}_{{\text{2peak}}}}$ following HIIT and CT training in the present study is in accord with findings from a previous study employing HIIT (+24%) and CT training (+19%) (Trapp et al. 2008). Taken together, these findings are in line with a report that increasing exercise intensity during short-duration exercise and ensuring that lower-intensity exercise duration exceeds 35 min are important factors for improving cardiorespiratory fitness (Wenger and Bell 1986).

The improved $\dot {V}{{\text{O}}_{{\text{2peak}}}}$ following HIIT and CT training in the present study was accompanied by improvements in aspects of cardiovascular function. Indeed, HIIT and CT training lowered resting HR by 5 bpm and 4 bpm, respectively. This is important as resting HR has been recognised as an independent risk factor for cardiovascular disease in women and is recommended to form part of the cardiovascular risk assessment (Perk et al. 2012). Moreover, HR was lower during submaximal walking after HIIT. This lowering of HR post training is likely linked to an increased cardiac stroke volume (Blomqvist and Saltin 1983). The lower resting systolic BP (by 5 mmHg) and MAP following CT training, is similar to a meta-analysis which reported a significant reduction in systolic BP (~3 mmHg) in normotensive individuals following aerobic exercise performed three to five times per week for 30–60 min (Cornelissen and Fagard 2005). It could be speculated that a reduction in BP may be more sensitive to exercise volume rather than intensity. This is of interest as a reduction of 5 mmHg in systolic BP has been estimated to reduce stroke, coronary heart disease and all-cause mortality by 14, 9 and 7%, respectively across the general population (Whelton et al. 2002). The reduction in systolic BP might be a function of a lower sympathetic and increased parasympathetic outflow, consistent with a lower resting HR, and/or increased muscular capillarisation and vascular remodeling with a resulting reduction in systemic vascular resistance (Andersen et al. 2010). These collective changes in cardiovascular function might have the potential to increase muscle oxygen (O_2_) delivery following HIIT and CT training which, along with potential improvements in mitochondrial biogenesis and function (Nordsborg et al. 2015), and muscle O_2_ extraction (Daussin et al. 2007), might account for the improved $\dot {V}{{\text{O}}_{{\text{2peak}}}}$ following HIIT and CT training in the present study.

Serum total [cholesterol], [HDL], total cholesterol/HDL ratio, [LDL], [triglycerides], fasting [glucose] and responses to the OGTT were unchanged in both training groups, which is consistent with some (Connolly et al. 2016; Trapp et al. 2008) but not all (Robinson et al. 2015) previous observations. Since the participants in the present study exhibited normal baseline values for these variables, this might account for the lack of training-induced changes. However, as explained by Gibala et al. (2014), the lack of change in blood [glucose] following HIIT in the present study may also be due to the reduced breakdown of muscle glycogen in type I fibers following HIIT in women compared to men. Indeed, the increased rate of glycogen breakdown and resynthesis following HIIT has reported to be important for improvements in insulin sensitivity (Gibala et al. 2014). In contrast, there was a small but significant decrement (−0.7 kg) in body mass following HIIT with no change in CT or CON groups. Although statistically significant, the 1% reduction in body mass is lower than the 5–10% reduction recommended for overweight and obese individuals to reduce their cardiovascular risk profile (Wilson et al. 1999). Therefore, the clinical relevance of this change in body mass after HIIT is likely to be small. The lack of change in fat mass of the present participants is in agreement with some (Keating et al. 2014) but not all (Trapp et al. 2008) previous studies on overweight adults following 12–14 weeks of HIIT.

To our knowledge, this is the first study to report similar improvements in visual learning and memory and verbal learning following self-paced HIIT and CT training, and improved verbal memory following self-paced HIIT but not CT, in premenopausal, inactive females. These improvements are in line with findings from a recent systematic review by Cox et al. (2015). Participation in high-intensity exercise has been associated with the upregulation of brain-derived neurotrophic factor (BDNF) which has been linked to the stimulation of the hippocampus and pre-frontal cortex leading to chronic improvements in cognitive function via neurovascular remodeling including neuro/synaptogenesis and angiogenesis (Hillman et al. 2008). In line with the similarities in mean training HR, this may help explain the similar improvements in cognitive function following HIIT and CT in the present study. An improvement in verbal memory was also found for the HIIT group following completion of the ISLTR (delayed recall) test. It should be acknowledged however that the ISLTR test is based on a possible score out of 12 which was attained by a number of individuals on their baseline visit and thus a ceiling effect was present. Despite this limit to sensitivity, the HIIT group still displayed a within-group improvement. However, the lack of equivalent effect in the CT group may partially result from the limited scope for improvement. Interestingly, baseline results of all cognitive tests employed in the present study were similar to, or better than, normative data provided by Cogstate of 341 healthy individuals aged 35–49, which could account for the lack of improvement in some of the tests. Therefore, while both CT and HIIT enhanced cognitive function, improvements were attained with a smaller time commitment following HIIT suggesting that this exercise modality might provide a more time-efficient exercise modality to enhance cognitive function compared to CT training, at least in inactive, premenopausal women.

Following training, well-being scores were only improved in CT. This corroborates previous studies reporting that mental well-being improves more with CT training compared to HIIT (Moses et al. 1989). However, although not significantly different, baseline scores were slightly higher for HIIT (52) compared to CT (49) and remained higher following the training intervention (HIIT = 54; CT = 52). In addition, post-intervention enjoyment (GEQ) was not different between training groups in this study. The similar levels of enjoyment in CT training and HIIT are in line with some previous studies (Heinrich et al. 2014), but conflict with other studies reporting greater enjoyment following HIIT (Kong et al. 2016) or CT training (Foster et al. 2015). These contrasting results could be due to discrepancies in the HIIT and CT exercise training protocols and the stage of the training intervention at which enjoyment levels were assessed. Indeed, as RPE was significantly higher for HIIT during the first 8 weeks of exercise, this may have affected enjoyment. Therefore, it is possible that if enjoyment was assessed during earlier time points in the intervention, between-training group differences may have occurred based on the differences in RPE. However, it is likely that the participants became accustomed to the higher intensity of exercise at the later stages of the intervention (+8 weeks) as no difference in RPE existed between training groups in the final 4 weeks, even though both groups significantly increased their power output. These findings suggest that, if inactive individuals are able to tolerate the initial higher perceived exertion during HIIT (8 weeks), they are likely to report similar levels of enjoyment to CT. This is important as enjoyment of exercise can predict adherence (Parfitt and Hughes 2009). Nonetheless, it should also be noted that, even though RPE was higher in the first 8 weeks of HIIT, no participants dropped out of this group due to the intensity with a single participant dropping out of the HIIT and CT training groups over the course of the intervention. This may partly be attributable to the fact that the participants were able to self-pace their workout during every training session.

Although the American College of Sports Medicine recommends 150 min of moderate or 75 min of vigorous physical activity per week to improve health (Garber et al. 2011), and while self-paced HIIT and CT training were similarly effective at improving some health markers, HIIT and CT training differed in their ability to improve other health-related variables in the current study. It should also be noted that, based on previous research which classified responders and non-responders using two times the typical error for results obtained from repeated $\dot {V}{{\text{O}}_{{\text{2peak}}}}$ tests separated by a week in recreationally active adults (20 years) (Bonafiglia et al. 2016), four participants from the HIIT group could be classed as non-responders or at least having a low sensitivity to HIIT. There were no non-responders following CT. Therefore, further research is required to assess whether combining these two training methods, or switching training methods (Bonafiglia et al. 2016), can produce greater health benefits compared to either training method completed independently. Additionally, it should be noted that testing periods were not timed in relation to the menstrual cycle and the social aspect of training was not controlled in relation to the number and interaction of participants during training which could increase variance in the reported results (Gibala et al. 2014). It is noteworthy that the 10–20–30 training concept, which was employed as the HIIT method in the current study, resulted in clinically-relevant improvements in established health markers despite the completion of only ~12 min cycling at a ≥90% self-perceived intensity per week. This is striking as the completion of high-intensity work was considerably lower than the recommended 75 min of vigorous activity per week to improve health (Garber et al. 2011). Moreover, the adaptations to HIIT were achieved from the completion of less total work done and for a lower time commitment. Collectively, our results indicate that both self-paced HIIT and CT training are effective interventions to improve established health markers in inactive middle-aged premenopausal women, but that self-paced HIIT is a more time efficient strategy to elicit many of the adaptations that can be achieved through conventional self-paced CT training. Ultimately, given that enjoyment was similar between groups, previously inactive middle-aged premenopausal women should be informed that both self-paced HIIT and CT are beneficial to health as long as the intervention-specific time commitments are adhered to.

In conclusion, 12 weeks of self-paced HIIT and CT cycle training were similarly effective at improving cardiorespiratory fitness, resting HR and RPP and cognitive function in inactive middle-aged premenopausal women which may be due, in part, to the similar mean HR between groups, with both groups training at a vigorous intensity. However, BP was lowered and well-being was only improved following CT training and submaximal exercise HR and total body mass were only lowered by HIIT, indicating that some health markers are more likely to be improved in a training-type-specific manner. Although participants reported similar levels of enjoyment and showed similar levels of adherence to both training methods, the adaptations to HIIT were achieved for the completion of less work and the commitment of less time compared to CT training. These findings support the use of self-paced exercise training methods (HIIT or CT) to improve the health profile of inactive middle-aged premenopausal women, as long as the intervention-specific time commitments are adhered to. These findings might have implications for exercise prescription for the improvement of clinically-relevant health markers in inactive middle-aged premenopausal women. While beneficial adaptations on health markers are clearly apparent, individuals unaccustomed to vigorous exercise, in particular older adults, should consult their doctor before starting an exercise program.

## Abbreviations

ANOVA: Analysis of variance
BMI: Body mass index
BP: Blood pressure
bpm: Beats per minute
cm: Centimetre
CON: Control
CT: Continuous training
CV: Coefficient of variation
GEQ: Groningen Enjoyment Questionnaire
HDL: High-density lipoprotein
HIIT: High-intensity interval training
HR: Heart rate
HR_peak_: Peak heart rate
ISLT: International shopping list task
ISLTR: International shopping list task delayed recall
kj: Work done
kg: Kilogram
MAP: Mean arterial pressure
min: Minute
ml: Millilitres
ml·kg^−1^·min^−1^: Millilitres per kilogram body mass per minute
mmHg: Millimetres mercury
LDL: Low-density lipoprotein
NCD: Non-communicable disease
OCL: One card learning task
OGTT: Oral glucose tolerance test
ONB: One back task
RPE: Ratings of perceived exertion
RPP: Rate pressure product
SD: Standard deviation
tAUC: Total area under the curve
TWOB: Two back task
$\dot {V}{{\text{O}}_{{\text{2peak}}}}$: Maximal oxygen uptake
W: Watt
WEMWBS: Warwick-Edinburgh mental well-being scale
